# Particle bombardment-mediated co-transformation of the *Cht-2* gene in wheat and the associated changes in defense mechanisms in transgenic plants infected with *Fusarium graminearum*

**DOI:** 10.1016/j.dib.2018.09.130

**Published:** 2018-10-16

**Authors:** Hanan A. Hashem, Raifa A. Hassanein, Ashraf H. Fahmy, Ahmed S. Ibrahim, Osama M. El Shihyh, Ebtesam A. Qaid

**Affiliations:** a Department of Botany, Faculty of Science, Ain Shams University, Abbassia, Cairo, Egypt; b Agricultural Genetic Engineering Research Institute, 9 Gamaa St., Giza, Egypt; c Department of Plant Physiology, Faculty of Agriculture, Cairo University, Egypt; d Department of Biology, Faculty of Science, Taiz University, Yemen

## Abstract

*Fusarium graminearum* is a major global pathogen of cereals and is considered the main causal agent of *Fusarium* head blight disease in wheat. Infection with *Fusarium graminearum* causes a significant reduction in crop yield and quality; therefore, it is very important to improve wheat pathogen resistance. In the present study, the plasmid pAHCht-2 harboring the *rice chitinase* (*Cht-2*) gene for pathogen resistance and the plasmid pAB6 containing the *gus* reporter and *bar* selectable marker genes were used for genetic transformation of immature embryo-derived calli of the Egyptian wheat cultivar Giza 164 using particle bombardment. Associated changes in defense mechanisms in the transgenic plants were investigated. The transgenic plants had significantly decreased total protein content, phenolic compounds and antioxidant enzyme activities (peroxidase and catalase), and significantly increased phenylalanine ammonia lyase and chitinase activities compared with non-transgenic plants under biotic stress conditions caused by *F. graminearum* infection. Our results show that activating a specific program of gene expression related to environmental stress conditions can reduce the cost of the stress on plant metabolism.

**Specifications table**

**Table:** 

Subject area	*E.g., Plant biology*
More specific subject area	*Plant Biotechnology, Plant physiology*
Type of data	*Table, text file, figure*
How data was acquired	*Microscope, survey, SEM, NMR, mass spectrometry, etc.; if an instrument was used, please give the model and make.*
Data format	*analyzed*
Experimental factors	*Transformation of wheat plant with Ch-2 gene*
Experimental features	the plasmid pAHCht-2 harboring the *rice chitinase* (*Cht-2*) gene for pathogen resistance and the plasmid pAB6 containing the *gus* reporter and *bar* selectable marker genes were used for genetic transformation of immature embryo-derived calli of the Egyptian wheat cultivar Giza 164 using particle bombardment. The presence and integration of transgenes were assessed by PCR analysis using specific primers for the *Cht-2*, *bar* and *gus* genes. The incorporation of the *Cht-2* gene into the genome of the transformants was confirmed by dot-blot analyses. The transformation efficiency (number of transgenic plants/number of embryos) was 6.01%. Additionally, associated changes in defense mechanisms in the transgenic plants were investigated.
Data source location	*Cairo/ Egypt*
Data accessibility	*All data included in the attached files*
Related research article	*N/A*

**Value of the data**

The objective of the present work was to produce pathogen-resistant wheat transformed with a rice chitinase gene (*Cht-2*) and to detect the associated changes in defense mechanisms in transgenic plants infected with *Fusarium graminearum* compared with non-transgenic plants. The particle bombardment method has been accepted as a breakthrough because genetic transformation with this method has become almost a routine process in many important crop species recalcitrant to transformation with other techniques. In fact, wheat has remained difficult to use in transgenic studies, mainly because of the lack of explants with high regeneration efficiency. We succeed to produce a genetically transformed wheat plants with increased pathogen resistant. The results also confirmed that genetic transformation of wheat plants with a rice chitinase gene (*Cht-2*) caused significant physiological changes including increases in chitinase and PAL activities compared with non-transgenic plants. In addition, although most of the transgenic lines inoculated with 20% *F. graminearum* culture filtrate had significantly elevated amounts of total soluble protein and increased CAT, POX and APX activities compared with non-inoculated plants, such increases were significantly lower than those detected in non-transgenic plants subjected to the same stress conditions, indicating that activating a specific gene expression program related to particular environmental stress conditions can reduce the cost of the stress on plant metabolism.

## 1 Data

The particle bombardment method has been accepted as a breakthrough because genetic transformation with this method has become almost a routine process in many important crop species recalcitrant to transformation with other techniques [1]. In fact, wheat has remained difficult to use in transgenic studies, mainly because of the lack of explants with high regeneration efficiency [2]. Immature wheat embryos are generally considered the optimum explants for plant regeneration in wheat, and have been widely used for transformation in various protocols [3], [4].

The objective of the present work was to produce pathogen-resistant wheat plants (*Triticum aestivum* L. cv. 164) transformed with a rice chitinase gene (*Cht-2*) and to detect the associated changes in defense mechanisms in transgenic plants infected with *Fusarium graminearum* compared with non-transgenic plants.

## 2 Experimental design, materials, and methods

### 2.1 Plant material and tissue culture

#### 2.1.1 Sterilization of immature caryopses

Immature caryopsis of the Egyptian wheat cultivar Giza 164 was collected approximately two weeks post anthesis. Immature grains were surface sterilized by soaking for 1 min in 70% (v/v) ethanol followed by 20% commercial Clorox (5.25% Sodium hypochlorite) supplemented with few drops of Tween 20, and washed five times with sterile ddH_2_O. Procedures used for infection and biolistic gene transformation.

#### 2.1.2 Isolation and culturing of the explant

Immature embryos were aseptically isolated with forceps under sterile conditions in the laminar air-flow hood. Fifty immature embryos were cultured with the scutellum side up onto the callus induction medium modified for wheat cell culture [5], [6]. Basically, it contains MS [7] medium salt, supplemented with 2 mg/L 2,4-D as a source of auxin, 0.15 g of L-Asparagine, 0.1 g of myo-inositol, 50 mg thiamine-HCL, 20 g sucrose and adjusted to 5.8 pH and solidified by 2.5 g/L phytagel. Callus induction medium was used for both infection and particle bombardment procedures. Immature embryos were cultured in the dark at 25 °C on the callus induction medium for one week for bombardment.

### 2.2 Wheat transformation

#### 2.2.1 Bacterial strain and genetic construct

##### 2.2.1.1 *E. coli* DH10β was used for bacterial transformation

**Fig. 1:**
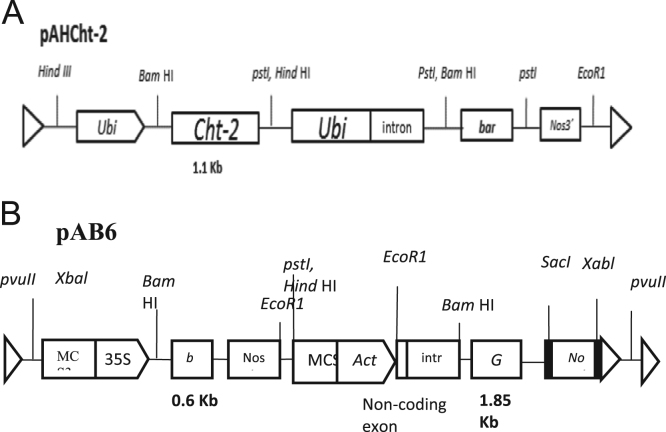
Schematic representation of plasmids pAHCht-2 (A) and pAB6 (B) used for co-bombardment.

Two different plasmids were used for the co-bombardment; the pAHCht-2 plasmid contains the *Cht-2* gene, which is a 1.1 kb *rice chitinase* class I under the transcriptional control of the maize ubiquitin promoter (Fig. 1A). The pAB6 plasmid contains *gus* (1.8 kb) gene (driven by rice Act1 intron promoter and the nopaline synthase *nos* terminator and the selectable marker / herbicide resistance *bar* (0.6 kb phosphinothricin acetyl transferase) gene (driven by cauliflower mosaic virus 35S promoter and the nopaline synthase *nos* terminator (El-Mangoury et al., 2006). The plasmid DNA was suspended in 10 mM Tris–HCl and 1 mM EDTA buffer (pH 8.0) at a concentration of 1 µg/µl.

##### 2.2.1.2 Transformation of plasmid DNA into *E. coli* competent cells

The highly efficient competent cells of *E. coli* (DH10β) for the transformation with plasmid DNA were prepared according to the method of Ausubal et al. [8] using ice-cold calcium chloride treatment.

##### 2.2.1.3 Plasmid purification

Plasmid purification was done through three main steps; growth of bacterial culture harboring the plasmid, lysis of bacteria and isolation of plasmid. Lysis by alkali compounds was done based on the method of Birnboim and Doly [9] and Ish-Horowicz and Burke [10].

Wizard ™Megapreps DNA purification System (Promega, USA) was used as large scale DNA purification system.

##### 2.2.1.4 Genetic transformation of wheat

Plant transformation was carried out by particle bombardment technique using the Biolistic® PDS-1000/He device (Bio-Rad, USA).

Immature embryo-derived calli of three Egyptian wheat cultivar Giza 164 were osmotically treated with 0.2 M mannitol and 0.2 M sorbitol for four hours before bombardment. Small calli were bombardment with 1.0 µm gold particles coated with pAHCht-2 and pAB6 plasmids. The distance between the particle holder and target was 6 cm and helium pressure was 1100 psi. Calli were remained for additional 16 h on the same osmotic treatment. Then calli were transferred to induction medium without osmotic medium to be recovered for additional one week.

Calli were then assayed by histochemical GUS activity assay. The remaining calli were transferred to selective medium supplemented with 3 mg/L phosphinothricin (PPT). Calli showing vigorous growth were sub-cultured twice every three weeks onto selection medium, and then transferred onto regeneration medium supplemented with 1.5 mg/L thidiazuron (TDZ) until emerging of shoots. Vigorous shoots were transferred to rooting medium with half strength MS medium.

### 2.3 Acclimatization

After development of a root system, regenerated putatively transgenic plants were then transferred to soil mixture; peat moss: sand: clay with a ratio (1:1:1); respectively, in small pots and covered with plastic pages, and then placed in a controlled growth chamber at 25 °C for 3 weeks, then were transferred to big pots and grown to maturity under greenhouse conditions. The seeds were then collected from grown booted plants.

### 2.4 Histochemical analysis

#### 2.4.1 Assay of β-glucuronidase (gus) activity

GUS assay was carried out as described by Jefferson et al. [11]. After recovery, Calli were incubated in X-Glue solution containing 1 mM (5-bromo-4-chloro-3-indolyl-β-D-glucuronide), 0.1% (v/v) Triton X-100, 20% methanol and 100 mM sodium phosphate buffer (pH 7.0) and 0.5 mM potassium ferricyanide. To compare transient and stable *gus* transformation, GUS analysis was done.

For observing transient *gus* expression, bombardment calli after 2 days on induction medium were dipped in GUS staining solution and was incubated at 37 °C for 2–3 days and then callus expressing *gus* photographed under the binocular stereomicroscope.

For observing stable *gus* expression, regenerated plants were assayed by dipping transformed plantlets into GUS staining solution. The reaction mixture was incubated at 37 °C for 2–3 days, chlorophyll was extracted from the tissue by incubation in 70% ethanol followed by 100% ethanol and regenerated plants expressing *gus* were photographed under the binocular stereomicroscope.

#### 2.4.2 Assay of bar expression analysis

Leaf painting assay was used to study the integration and expression of the *bar* gene in T0 plants according to Cho et al. [12]. 0.2% of Liberty solution, containing 0.1% (v/v) Tween-20, was applied to leaf sections using a cotton swab. Resistance to the herbicide solution was examined after seven days of application by observing of leaf necrosis.

### 2.5 Detection of transformed DNA in genetically modified samples using Polymerase Chain Reaction (PCR)

Total genomic DNA was isolated from wheat leaves using a cetyltriethyl-ammonium bromide (CTAB) extraction method [13]. The PCR analysis was used to confirm the presence or the absence of the three transgenes (*cht-2*, *bar* and *gus*) in the transformed plants. The PCR analysis was performed on Bio-RAD T100™ Thermal cycler. DNA electrophoresis was performed in Scie-Plas cell. The specific primers used to amplify of *cht-2* gene were 5׳TAAGGATGTCGACGCCGAGAGGGG3׳ and 5׳CGTCAGTCCTCATCACTGCTCCGG3׳ (1100 bp). The forward and reverse primers employed for detection of *bar* gene were 5׳ CAG ATC TCG GTG ACG GGC AGG C3`and 5` CCG TAC CGA GCC GCA GGA AC -3` (443 bp); and for *gus* gene were 5`AGT GTA CGT ATC ACC GTT TGT GTG AAC 3`and 5`AGT GTA CGT ATC ACC GT TTG TGT GAA C3` (1050 bp).The PCR program profile for three genes was done as follows: initial denaturation at 95 °C for 5 min, followed by 30 cycles at 94 °C for 30 s, annealing for 30 s and 72 °C for 1 min and finally, an additional elongation step was performed for 5 min at 72 °C. the annealing temperature for the amplification of *cht-2*, *bar* and *gus* genes were 61.3 °C, 58 °C and 62 °C, respectively. The PCR reaction mixture contained 50 ng of template DNA, 0.5 µM of each primer, 10 mM of dNTPs, 2.5 mM of MgCl_2_, PCR buffer and *Taq* polymerase in a volume of 25 µl. The amplified products were electrophoretically resolved on a 1% agarose gel in Tris-acetate EDTA (TAE) buffer.

### 2.6 Dot-blot hybridization analysis

PCR products from transgenic plants, non-transgenic plants (negative control) and pAHCht-2 (positive control) were denatured and neutralized with 0.4 M NaOH, 10 mM EDTA, incubated at 96 °C for 10 min, then rapidly cooled in ice. Using a dot-blot manifold, samples were spotted onto pre-soaked nitrocellulose membrane cover two pieces of Whatman paper. Membrane was cross-linked under UV light. Hybridization was performed at 45 °C for overnight in a buffer containing 5× denhardt׳s solution, 6× sodium chloride-sodium citrate (SSC), 0.5% sodium dodecyl sulfate (SDS) and 50% (v/v) deionized formamide and followed by the addition of pAHCht-2 as probe. Membrane was washed twice at room temperature in 2× SSC/ 0.1% SDS for 5 min followed by two washes in 0.1× SSC/ 0.1% SDS for 20 min at 70 °C. Direct procedure and detection system was carried out by Biotin Chromegenic Detection Kit (Thermo Scientific). Blot was washed and product detection conducted by the addition of BCIP/NBT solution, then the blot was exposed to photography.

### 2.7 Physiological determination

Seven lines from transformed callus that had high regeneration rate were chosen. Each line was divided into two groups: first group (non-inoculated plants) of plantlets were transferred into rooting medium (1/2 MS), while the second group (inoculated plants) of plantlets were also transferred into rooting medium (1/2 MS supplemented with 20% of *F. graminearum* culture filtrate). Non-transgenic plantlets were transferred to rooting medium as control for first group. Another group of non-transgenic plantlets were transferred to rooting medium containing 1/2 MS supplemented with 20% of *F. graminearum* culture filtrate as control for the second group. Both treated and control groups were kept at 24 °C under 16 h / 8 h light/ dark cycle for two weeks. Physiological parameters were determined (total soluble proteins; the activity of certain antioxidant enzymes; PAL activity, chitinase activity, proline; total phenol; flavonoid contents) after two weeks in non-inoculated and inoculated state of both non-transgenic and transgenic plants.

Statistical analysis of physiological parameters was analyzed using Duncunˈs multiple range test at 5% probabilities according to SPSS program version 17.

#### 2.7.1 Antioxidant enzymes

##### 2.7.1.1 Peroxidase (POX) (EC 1.11.1.7)

POX activity was assayed by the method of Velikova et al. [14]. The activity of POX was calculated from the rate of formation of guaiacol dehydrogenation product (GDHP) using the extinction coefficient of 26.6 mm^−1^ cm^−1^, and the activity was expressed as μM GDHP / min / g fresh weight.

##### 2.7.1.2 Catalase (CAT) (EC 1.11.1.6)

CAT activity was measured according to Aebi [15]. The reaction mixture contained 50 mM potassium phosphate buffer (pH 7.0), 0.1 ml enzyme extract and 0.035% H_2_O_2_. The activity of CAT was calculated based on the decomposition of H_2_O_2_ measured as a decline in the absorbance at 240 nm. The activity was calculated using the extinction coefficient of 40.84 mm^−1^ cm^−1^, and expressed as μM H_2_O_2_ reduced / min / g fresh weight.

##### 2.7.1.3 Ascorbate peroxidase (APX) (EC 1.11.1.11)

The reaction mixture contained 0.5 mM ascorbic acid, 0.1 mM EDTA and 0.1 mL of enzyme extract. The reaction was initiated when adding 1.5 mM H_2_O_2_. The absorbance of the reaction mixture was measured at 290 nm 2 min after H_2_O_2_ was added. The activity of APX was calculated using the extinction coefficient of 3.98 mm^−1^ cm^−1^and the activity was expressed as μM ascorbate oxidized / min / g fresh weight [16].

#### 2.7.2 Phenylalanine ammonia lyase (PAL) (EC 4.3.1.5)

Shoot tissues (0.15 g) were homogenized in 1.5 ml of ice-cold 0.1 M sodium borate buffer, pH 7.0 containing 1.4 mM of 2-mercaptoethanol and 0.1 g of insoluble PVP. The homogenate was centrifuged at 12,000*g* for 30 min and the supernatant was collected for enzyme assay. PAL activity was determined based on the rate of conversion of L-phenylalanine to *trans*-cinnamic acid at 290 nm [17]. The activity of PAL was calculated using its extinction coefficient of 9.630 mM^−1^ cm^−1^ and the activity was expressed as μM *trans*-cinnamic acid synthesized / min / g fresh weight.

#### 2.7.3 Determination of total soluble protein

Total soluble proteins were extracted from the samples. The method described by Guy et al. [18], the tissue ground in liquid nitrogen was extracted with buffer containing ice-cold 50 mM Tris–HCl, pH 7.5; 2 mM EDTA and a 0.04% (v/v) 2-β-mercaptoethanol. The homogenate was centrifuged at 14000 rpm for 20 min at room temperature. Protein was determined using the dye-binding assay of Bradford [19] with bovine serum albumin as a standard.

#### 2.7.4 Measurement of free proline content

The proline content in the shoots and roots of two cultivars was determined according to the method of Bates et al. [20]. Fresh weight of tissue (0.5 g) was ground in mortar and pestle with 10 ml of 3% sulphosalicylic acid then the homogenate was filtered. Two ml of the filtrate was mixed with 2 ml of ninhydrin reagent (1.25 g ninhydrine in 3 ml glacial acetic acid and 20 ml 6 M phosphoric acid with agitation, until dissolved) and 2 ml of glacial acetic acid. Four ml of the toluene was added to the mixture, mixed vigorously with a test tube stirrer for 15–20 s. The chromophore containing toluene was aspirated from the aqueous phase and absorbance read at 520 nm. The results were expressed as μg/g fresh weight.

#### 2.7.5 Determination of free phenol content

Accurately weighed of tissue (0.1 g) was extracted with chilled 1.5 ml 80% methanol with 1% HCl. The extract was centrifuged at 3000*g* for 10 min. Supernatant was used to determine phenol content. Each extract was prepared freshly for the analysis to prevent any degradation. Free phenol content in the extract of wheat cultivar treated and non-treated were immediately determined using the Folin-Ciocalteu׳s method by Hung et al. [21]. The content of phenolics in the extracts was expressed in terms of gallic acid equivalent (mg of GA / g of dry tissue).

#### 2.7.6 Determination of flavonoid content

The flavone compounds were extracted according to the method reported by Abeysinghe et al. [22]. The content of flavonoids in the examined plant extracts was determined using spectrophotometric method (Quettier et al., 2000). The absorbance was determined using spectrophotometer at 415 nm. The concentration of flavonoids was read (mg / ml) on the calibration line; then, the content of flavonoids in extracts was expressed in terms of rutin equivalent (mg of RU / g of dry tissue).

#### 2.7.7 Chitinase assay (EC 3.2.1.14)

One gram of fresh leaves was homogenized with a pestle and mortar in liquid nitrogen and the frozen powder was suspended in one ml ice-cold 10 mM sodium acetate buffer (pH 5.0) containing PVP and 20 mM sodium ascorbate. The homogenate was centrifuged at 13,000 rpm for 15 min two rounds. The supernatant was transferred to a fresh tube and kept at −20 °C. Chitinase activity was measured by the release of N-acetyl glucosamine (NAG) using colloidal chitin, which was prepared according to Berger and Reynolds [23], as substrate according to the method of Reissig et al. [13]. The hydrolysis products were detected using p-dimethyl aminobenzaldehyde (p-DMAB). The absorbance was recorded at 585 nm using N-acetylglucosamine as standard. One unit was defined as the amount of enzyme that produced 1 µmol of reducing sugar corresponding to N-acetyl-D-glucosamine (NAG) in one min. The chitinase activity was expressed as µmole/min/g fresh weight.
