# Supplementary material for: Particle bombardment-mediated co-transformation of the Cht-2 gene in wheat and the associated changes in defense mechanisms in transgenic plants infected with Fusarium graminearum
Source: Data Brief. 2018 Oct 16;21:1111–8. doi: 10.1016/j.dib.2018.09.130 (PMC6230962; doi:10.1016/j.dib.2018.09.130)
Supplement: Supplementary file 1 — Supplementary material [file mmc1.docx]

**Conflict of Interest**

The authors declare that they have no conflict of interest.
